# The management of adult appendicitis during the COVID-19 pandemic: an interim analysis of a UK cohort study

**DOI:** 10.1007/s10151-020-02297-4

**Published:** 2020-07-15

**Authors:** H. Javanmard-Emamghissi, H. Boyd-Carson, M. Hollyman, B. Doleman, A. Adiamah, J. N. Lund, R. Clifford, L. Dickerson, S. Richards, L. Pearce, J. Cornish, S. Hare, S. Lockwood, S. J. Moug, G. M. Tierney, The COVID: HAREM (Had Appendicitis, Resolved/Recurred Emergency Morbidity/Mortality) Collaborators Group, H. Javanmard-Emamghissi, H. Boyd-Carson, M. Hollyman, B. Doleman, A. Adiamah, J. N. Lund, R. Clifford, L. Dickerson, S. Richards, L. Pearce, J. Cornish, S. Hare, S. Lockwood, S. J. Moug, G. M. Tierney, Nikhil Kulkarni, Isabel Pereira, Sarah Barlow, Sarannga Vanniasegaram, Natalie S. Blencowe, Benjamin E. Zucker, Abigail Tyer, Marianne Hollyman, Angeliki Kosti, Thomas Badenoch, Sarah Wheatstone, Mariam Jaffer, Hannah Gerretsen, Rahul Menon, Muhammad S. Sajid, Lauren Kennedy, Ahmed Malik, Abeer Nada, Kausik Ray, Mansoor Khan, Massimo Varcada, Farid Froghi, Amjad Khalil, Demetra Kyprianou, Nila Tewari, Diwakar Ryali Sarma, Mariam Baig, Sumit Sood, Evonne Yu Wen Ng, Vincent Ng, Thomas Shortland, Gabriel Marangoni, Saboor Khan, Jawad Ahmad, Steven Brown, Arslan Pannu, Elizabeth Gemmill, Hannah Boyd-Carson, Philip Herrod, Satnam Singh Shari, Mohammed Jibreel Suliman Mohammed, Vijay Narbad, Nabih Hanbali, Anisa Kushairi, M. A. Matthew, Candice Downey, Amro Alamassi, Tim Wheatley, Katy Emslie, Bruno Alcocer, Simon Lau, Richard Morgan, Tanzeela Gala, Sherif Ibrahim, Mina Stephanos, Reda Mithany, Mostafa Abdelkarim, Gautham Venkatesan, Ahmad Aqsalan, John Taylor, Matthew Fok, Arjun Kattakayam, Kunal Rajput, Katherine Bevan, Hyun-Kyung Kim, Laylan Salih, Regina Sabaratnam, Mihaela Creanga, Adil Shafi, Jennifer Law, Mohammed Elniel, Matthew Walmsley, Shruti Ayyar, Julie Cornish, Nicola Reeves, Nicholas Mowbray, Issac Mayo, Ezzat Chohda, William Mccaughran, Emma Beck, Sowmya Garikipati, Bryony E . Lovett, Firas Alkistawi, Chris Hadjitoffi, Aaliya Uddin, Panna K. Patel, Siddhartha Handa, Jessica Parker, Dawn Littlehales, Ajay P . Belgaumkar, Bankole Oyewole, Prabhat Narayan, Zain Elahi, Andrew Gaukroger, Declan Francis Joseph Dunne, George Emilian Nita, Ryan David Baron, Dana Sochorova, Peter Szatmary, Sukhpreet Ak Gahunia, Amy Jayne Thomas, Kulbir Singh Mann, Malcolm Mcfall, Nicholas Farkas, Hussam Siddig, John Camilleri-Brennan, Duncan Rutherford, Michael Wilson, Eleanor Massie, Kieran Mcgivern, Jennifer Mcguckin, Connor Mckee, Spyros Marinos-Kouris, Emanuele Gammeri, Nikhil Patel, Giulia Cillo, Alexander James Baldwin, Tania Magro, Kandaswamy Krishna, James Olivier, Ngozi Anyaugo, Ken Philip, Lyndsay Pearce, Azzam Al-Amin, Michael Thomas, Ian Anderson, Robert Clark, Gillian Tierney, Hannah Javanmard-Emamghissi, Carla Hope, Arjun Gowda, Dana Photiou, Francesca Malcolm, Prita Daliya, Zoe Chia, Najam Husain, Pradeep Thomas, Tomas Urbonas, Daniel Centea, Susan Moug, Christopher Brown, Mari-Claire Mcguigan, Carly Bisset, Abigail Ingham, Norman Galbraith, Rachael Clifford, Luke Dickerson, Sonia Lockwood, Judith Johnston, Ashish K . Shrestha, Anang Pangen, Charannya Balakumar, Sara Iqbal, Samip Prakash, Jaideep Rait, Anreea Hanu, Richard Guy, Talal Majeed, Robert Young, Sarah Shamim, Mina Mesri, Roshani Patel, Sophia Lewis, Adesuwa Theresa Eigbadon, Dixa Thakrar, Evangeline Karamitsou, Yetunde Oyeyipo, Uqba Nadeem, Sibusiso Ndlovu, Angela Fnshawe, Nikola Henderson, Christopher Payne, Darren Porter, Adam Brooks, Rachel Xue Ning Lee, Jamaal Jackson, Alastair James Morton, Olamide Ebunoluwa Oyende, Dawit Worku, Amanda Koh, Trisha Kanani, James Blackwell, Melissa Shaw, Christopher Lewis-Lloyd, Lauren Blackburn, Alfred Adiamah, Shafaque Shaikh, Mudassar Ghazanfar, Mootaz Elhusseini, Amir Abdelhamid, Jonathan Eley, Ahmed Nassar, Eriberto Farinella, Zeeshan Mahmood, Tania Policastro, Richard J . McGregor, Dimtrios Damaskos, Maria Drogouti, Zofia Tuharska, John Matthew Bennett, Ramez Antakia, Robert O’neill, Richard Hardwick, Nicola Fearnhead, Athanasios Xanthis, Fanourios Georgiades, Victoria Hudson, James Ashcroft, Arminder Singh, Laura Osborne, Ondrej Ryska, Beatrix Weber, Frederick Searight, Calum McCoss, Mark Bignell, Rikhilroy Patel, Giles Bond-Smith, Christopher Lewis, Gethin Williams, Harriet Whewell, Laurie Smith, Rucira Ooi, Anna Powell-Chandler, Alethea M . Tang, S . K. Richards, D. B. Thompson, Jonathan Van Dellen, Victor Alberto, Shahram Shirazi, Hossein Arang, Nabila Rahman, Eimear Monaghan, Kristine Dodds, Olaitan Babalola, Pascal Airhunmwunde, Imran Alam, Kelvin Wang, Fedder Artemis, Imeshi Wijetunga, Thomas Kidd, Keshav Nambiar, Cho Ee Ng, Toni Collier, Basil Ibrahim, Khizar Khan, Kumuthan Sriskandarajah, Theo Pelly, Joseph Vance-Daniel, Pawan Dhruva Rao, Kellie Bateman, Ana Gavrila, Muhammad Imran Aslam, Verda Amin, Richard Wilkins, Shahbaz Zafar, Charalampos Konstantinou, Sian Mcdonald, Annalie Baker, Amy Fardie, Arnold Hill, Josh De Marchi, Sorcha O’Grady, Gemma Faulkner, Hema Sekhar, Marta Martinez-Iglesias, Cameron Alexander, Eloise Lawrence, Graham Williams, Swati Bhasin, Rajesh Yagati Satchidanand, Chamindri Weerasinghe, Ian Dorrington, Aloka Liyanage, Ayesha Mian, Mihai Paduraru, Seshu Bylapudi, Krystian Pawelec, Milin Rao, Cleo Kenington, Sarah Hudson-Phillips, Zac Vinnicombe

**Affiliations:** 1 Royal Derby Hospital, University of Nottingham at Derby, Derby, UK; 2 Royal Derby Hospital, Derby, UK; 3 Musgrove Park Hospital, Taunton, UK; 4 NIHR Nottingham Digestive Disease Biomedical Research Centre, Nottingham University Hospitals NHS Trust, Nottingham, UK; 5 The Countess of Chester, Chester, UK; 6 Royal United Hospital, Avon, Bath, UK; 7 Salford Royal NHS Trust, Salford, UK; 8 Cardiff Royal Infirmary, Cardiff, UK; 9 Medway Maritime Hospital, Kent, UK; 10 Bradford Royal Infirmary, Bradford, UK; 11 Royal Alexandra Hospital, Paisley, UK

**Keywords:** Appendicitis, COVID-19, Non-operative, Antibiotics, Appendicectomy

## Abstract

**Background:**

Acute appendicitis (AA) is the most common general surgical emergency. Early laparoscopic appendicectomy is the gold-standard management. SARS-CoV-2 (COVID-19) brought concerns of increased perioperative mortality and spread of infection during aerosol generating procedures: as a consequence, conservative management was advised, and open appendicectomy recommended when surgery was unavoidable. This study describes the impact of the first weeks of the pandemic on the management of AA in the United Kingdom (UK).

**Methods:**

Patients 18 years or older, diagnosed clinically and/or radiologically with AA were eligible for inclusion in this prospective, multicentre cohort study. Data was collected from 23rd March 2020 (beginning of the UK Government lockdown) to 1st May 2020 and included: patient demographics, COVID status; initial management (operative and conservative); length of stay; and 30-day complications. Analysis was performed on the first 500 cases with 30-day follow-up.

**Results:**

The patient cohort consisted of 500 patients from 48 sites. The median age of this cohort was 35 [26–49.75] years and 233 (47%) of patients were female. Two hundred and seventy-one (54%) patients were initially treated conservatively; with only 26 (10%) cases progressing to an operation. Operative interventions were performed laparoscopically in 44% (93/211). Median length of hospital stay was significantly reduced in the conservatively managed group (2 [IQR 1–4] days vs. 3 [2–4], *p* < 0.001). At 30 days, complications were significantly higher in the operative group (*p* < 0.001), with no deaths in any group. Of the 159 (32%) patients tested for COVID-19 on admission, only 6 (4%) were positive.

**Conclusion:**

COVID-19 has changed the management of acute appendicitis in the UK, with non-operative management shown to be safe and effective in the short-term. Antibiotics should be considered as the first line during the pandemic and perhaps beyond.

## Introduction

Acute appendicitis (AA) is the most common general surgical emergency worldwide [1]. The lifetime risk of developing AA is 6.7% and 8.6% in females and males respectively [2]. More than 30,000 appendicectomies are performed in England alone each year [3]. Mortality from uncomplicated AA is extremely low at 0.1%; however, mortality increases with delay in presentation [1]. The risk of appendix rupture increases significantly from 36 h after onset of symptoms [4]. Gangrenous appendicitis occurs in 10% of patients, and perforation or abscess is seen in up to one fifth, and both are associated with increased complications [5].

In the UK, operative intervention within 48 h of presentation is recommended for AA [6]. Laparoscopic appendicectomy offers clear advantages over open appendicectomy including less postoperative pain, fewer surgical site infections, decreased length of hospital stay (LOS), and quicker return to normal function [7], and accounts for around 94% and 98% of appendicectomies performed in males and females respectively [8].

There is a growing evidence base that AA not complicated by gangrene or perforation can be managed without surgery [9, 10], is associated with shorter time away from work or education, significantly lower overall complication rate at 5 years after the episode of AA and is cheaper [11, 12]. However, AA can return after successful non-operative management [9]. Despite these considerations, operative treatment remains the first line treatment for nearly all cases of AA in the UK; management with antibiotics usually reserved for those presenting with AA complicated by phlegmon or abscess [6, 8, 13].

SARS-CoV-2 (COVID-19) brought widespread concerns of the spread of infection during aerosol generating procedures (AGPs) such as surgery, and particularly, laparoscopic surgery [14, 15]. In addition, research into COVID positive patients having surgery reported high mortality rates even following minor procedures [16]. Compounding this, there was a lack of personal protective equipment (PPE) for surgeons at the start of the pandemic [17]. Consequently, conservative management with antibiotics was recommended early in the pandemic by UK surgical Royal Colleges as first-line treatment for acute uncomplicated AA. To minimise aerosol generation, open surgery was recommended over laparoscopic surgery when surgery was required [15, 17–20]. Computerised tomography (CT) scan was recommended for diagnosing AA and the exclusion of perforation or other pathology presenting with right iliac fossa pain [20].

The impact of COVID-19 and the impact of recommendations on surgical practice in the UK have not yet been fully analysed. An early evaluation of any changes in standard UK practice should be performed to assess the safety of any move away from first line operative management of AA. This will inform practice during the rest of the COVID-19 pandemic, and potentially beyond. There is the opportunity to observe if the safe and efficient conservative management of AA previously seen in randomised controlled trials and meta-analysis in Europe and the USA is generalisable in the UK [21–26]. This interim analysis of our study aims to capture the management of AA during the first few weeks of the COVID-19 pandemic lockdown in the UK and to assess the 30-day outcomes [27].

## Materials and methods

### Study design

A prospective multicentre study on patients aged ≥ 18 years diagnosed either clinically and/or radiologically with AA in a secondary care setting was carried out. Data was collected from patients presenting from the date of the UK Government COVID-19 lockdown on 23rd March 2020. Study registration was delivered by the local principal investigator at each site as either a clinical audit or service evaluation. We collected routine, anonymised data that did not influence clinical care and published the protocol [27].

### Outcomes

The primary aim of this interim analysis was to report on the initial management of patients diagnosed with AA from the start of the UK lockdown. Outcomes included conservative or operative management, surgical approach (open or laparoscopic), COVID status, Personal Protective Equipment (PPE) usage, imaging modality (CT scan; ultrasound[USS]), interventional radiology (IR) drain placement, admission to critical care [Level 2 (High Dependency Unit) or 3 (Intensive Care Unit)], 30-day complication rate, mortality, and length of stay (LOS).

Conservative management was defined as initial treatment with antibiotics and/or IR drainage (i.e., not straight to surgery). Acknowledging that the IR drainage group and patients with simple appendicitis are different patient populations, when statistical analysis was performed a comparison was made between conservative management group (IR drain and antibiotics) versus operative group, and antibiotics alone versus operative group. Failure of conservative management occurred when conservative management changed to surgery after ≥ 2 days after initial assessment. Patient demographics and outcomes were analysed in intention to treat by planned initial conservative management, even if they progressed to surgery later, and those started laparoscopically, even where converted to open intraoperatively, were analysed in the laparoscopic group. LOS is the number of days in hospital during the primary admission and is expressed as median.

### Site recruitment

All hospitals in the UK that provide emergency care for patients diagnosed with AA were eligible to participate. Publicity for the project was supported by The Association of Surgeons of Great Britain and Ireland (ASGBI) and the Royal College of Surgeons of England (https://www.rcseng.ac.uk/coronavirus/rcs-covid-research-group/). Regional research collaboratives and social media (@covidharem) aided trainee-led recruitment of sites.

### Data collection

Patients presenting through the emergency department and surgical assessment units were screened by the local teams as per the inclusion criteria. Confirmation of local approval allowed collaborators to enter fully anonymised data to Research Electronic Data Capture (REDCap, www.project-redcap.org). The database was developed and maintained by the Major Trauma Team at Nottingham University Hospitals, UK.

In the interests of providing evidence to help clinicians with decision making going forward during the pandemic, we have performed an interim analysis of the first 500 patients submitted with 30-day follow-up.

### Statistical analysis

The study was done according to Strengthening the Reporting of Observational studies in Epidemiology (STROBE) guidelines for observational studies. Descriptive data was reported as median [interquartile range (IQR)] or number/total (%) as appropriate. For all outcomes, proportions were reported as the number of events/total patients with data due to missing data for some outcomes. When comparing two groups with nominal outcomes Chi-squared or Fisher’s exact test was used for inferential testing. For continuous, non-normal data the Mann–Whitney *U* test was used with *p* < 0.05 regarded as the level of statistical significance. Statistical analysis was performed using SPSS Version 26 (IBM, www.ibm.com/uk-en/analytics/spss-statistics-software) and Stata Version 16.1 (StataCorp, www.stata.com).

## Results

### Patient cohort

**Fig. 1:**
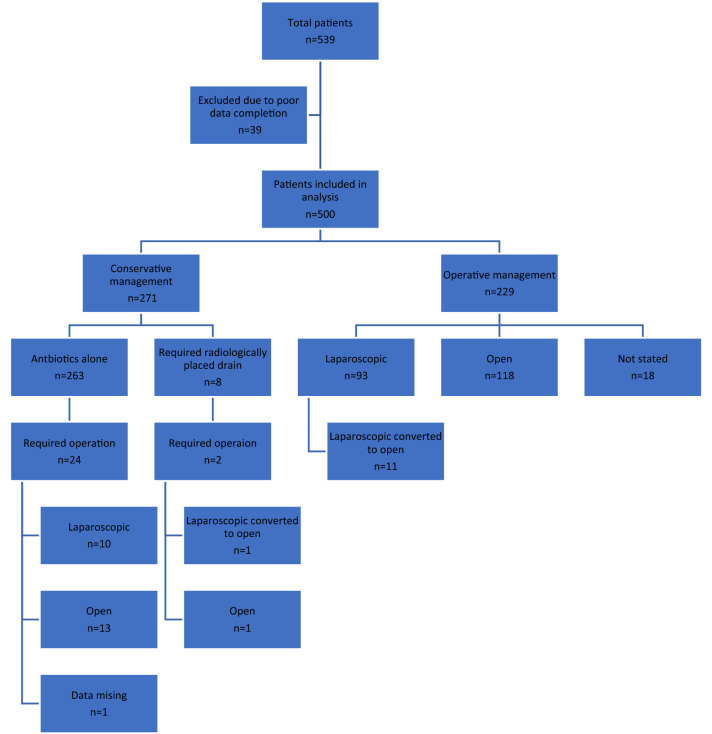
Study flow chart

From 23rd March 2020 to 1st May 2020, 539 patients were entered from 50 sites across the UK (Fig. 1). Participating sites were included in this analysis if their data achieved 95% completion, leaving 500 patients from 48 sites. The median age of this cohort was 35 [26–49.75] years and 233 (47%) of patients were female. Other demographics are displayed in Table 1.

**Table 1:** Participant characteristics at time of diagnosis and initial management of acute appendicitis

Event/total (%)*	Total (*n* = 500)	Operative management (*n* = 229)	Initial conservative management (*n* = 271)			
			Antibiotics and IR drain (*n* = 271)	*P* value	Antibiotics alone (*n* = 263)	*P* value
Age (years) median, range	35 [26–49.75]	37 [28.5–52]	34 [25–48]	0.08	34 [25–47]	0.04
Female	233/500 (47)	99/229 (43)	134/271 (49)	0.18	132/263 (50)	0.13
Body Mass Index kg/m^2^						
< 20	22/479 (5)	11/222 (6)	11/257 (3)	0.07	11/249 (4)	0.09
20–25	186/479 (39)	82/222 (37)	104/257 (41)		100/249 (40)	
25–30	165/479 (34)	68/222 (31)	97/257 (38)		94/249 (38)	
30–35	74/479 (15)	45/222 (20)	29/257 (11)		29/249 (12)	
35 +	32/479 (7)	16/222 (8)	16/257 (6)		15/249 (6)	
Rockwood score						
Not frail (1–3)	478/500 (96)	220/229 (96)	258/271 (95)	0.87	250/263 (95)	0.84
Pre-frail (4)	14/500 (3)	6/229 (3)	8/271 (3)		8/263 (3)	
Frail (5–9)	8/500 (2)	3/229 (1)	5/271 (2)		5/263 (2)	
Comorbidities						
None reported	450/499 (90)	207/229 (90)	243/270 (90)	1	236/262 (90)	1
Diabetes	18/499 (3)	9/229 (4)	9/270 (3)	0.81	9/262 (3)	0.81
COPD	9/498 (2)	4/228 (2)	5/270 (2)	1	5/262 (2)	1
Myocardial infarction	15/498 (3)	8/313 (3)	7/270 (3)	0.61	7/262 (3)	0.61
Immunosuppressed	11/499 (2)	3/229 (1)	8/270 (3)	0.24	7/262 (3)	0.35
Active cancer	4/499 (0.7)	3/313 (1)	1/270 (0)	0.3	1/262 (0.4)	0.34
Dementia	4/499 (0.7)	2/229 (0.6)	2/270 (0.8)	0.87	2/262 (0.8)	1
Imaging						
No Imaging	77/500 (15)	35/229 (15)	42/271 (16)	0.95	42/263 (16)	0.9
CT scan	353/500 (71)	174/229 (76)	179/271 (66)	**0.018**	171/263 (65)	**0.01**
USS	86/500 (17)	26/229 (11)	60/271 (22)	**0.002**	60/263 (23)	**0.001**
CT scan and USS	16/500 (3)	6/229 (2)	10/271 (3)	0.61	10/263 (4)	0.61
Admission COVID swab						
Positive	6/159 (4)	3/91 (3)	3/68 (4)	1	3/64 (5)	0.69
Negative	153/159 (96)	88/91 (97)	65/68(96)		61/64 (90)	
Not performed	341/500 (68)	138/229 (60)	203/271(75)	** < 0.001**	199/263 (76)	** < 0.001**
Length of stay (days) median, range	3 [1–4]	3 [2–4]	2 [1–4]	** < 0.001**	2 [1–4]	** < 0.001**
Managed without admission	57/483 (12)	9/223 (4)	48/260 (18)			** < 0.001**

Analysis is separated into operative group versus antibiotics and interventional radiological placed drain (IR drain) and operative group versus antibiotics alone. Continuous data is presented as median [IQR]; *p* values calculated by Mann–Whitney−*U*. Categorical data are presented as number/denominator (percentage); *p* values calculated by *χ*2 or Fisher’s exact test as appropriate

The bold typeset in the table highlights statistical significance

*COPD *Chronic obstructive pulmonary disease; *CT *Computed tomography, *USS *Ultrasound scan, *IR *Interventional radiology placement of

*Unless otherwise indicated

### Imaging

The majority of patients (425, 85%) had imaging to aid diagnosis. CT was the most commonly performed (353, 71%) throughout all ages in this study, including females under 40 years with no difference in frequency of use by gender (Table 2). An ultrasound scan was performed in 86 (17%), with females more likely to undergo ultrasound than males (76/233 vs. 10/267, *p* < 0.001).

**Table 2:** Computed tomography scans performed by age group

Age group	Computed Tomography (CT) scan	Females	Males	*P* value
18–39 years	165/300 (55)	74/144 (51)	91/156 (58)	0.23
40–59 years	118/128 (92)	46/52 (88)	72/76 (95)	0.07
60 years and above	70/72 (97)	36/37 (97)	34/35 (97)	1

There was a significant difference between age groups in CT scans performed (*p* < 0.001). Number and total (%) within each age group category;* p* values calculated by *χ*^2^

### Management approach

Two hundred seventy-one (54%) patients were initially managed conservatively, while the remaining 229 patients (46%) had a plan for surgery within the first day of admission (Fig. 1). Comparison of the initial conservative versus operative group found no difference between the two groups by sex, frailty score, comorbidities or smoking status (Table 1). Patients in the antibiotics alone group were younger than the operative group (median age 34 [25–47] years versus 37 [28.5–52] years, *p* = 0.04).

Within the initial conservative group, 263 (97%) received solely antibiotic therapy with 5 (2%) having an IR drain placed to manage an associated abscess on primary admission; 3 patients requiring an IR drain due to abscess following failed antibiotic management (5, 7 and 8 days after presentation). Failed conservative management occurred in 26 (10%), who went on to have surgery: 2 of these patients had previously had IR drainage. The decision that conservative management had failed and an operation was required, was made between 2–29-day post initial presentation, with 58% (15) of the decisions being made on the 2nd day after admission.

Within the operative group (*n* = 211), the majority had an open procedure 56% (118) versus 44% (93) laparoscopic, with a conversion to open reported in 11 patients (5%).

**Fig. 2:**
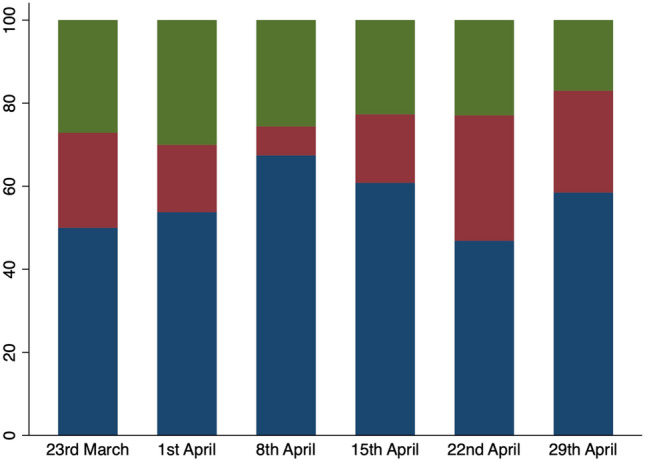
Percentage of patients treated nonoperatively (blue), with laparoscopic appendicectomy (red), and open appendicectomy (green) on a week by week basis during the pandemic

Figure 2 displays the week by week proportion of patients managed non-operatively and operatively (Fig. 2). During the first week of lockdown 35/74 (47%) of patients were initially managed conservatively, and of those who proceeded to operation, 19 (26%) had laparoscopic surgery and 22 (30%) had open surgery. By the third week of lockdown conservative management peaked at 64% (58/90).

### Patient outcomes and 30-day follow-up

LOS in the conservatively managed group, whether patients were given antibiotics alone or IR drain placed, was significantly less than in those who had an operation (2 [1–4] days vs. 3 [2–4] days, *p* < 0.001, Table 1). LOS was significantly lower in the laparoscopic appendicectomy group than the open appendicectomy group (2 [2–4] days vs. 3 [2–5] days, *p* < 0·012, Table 3).

**Table 3:** Comparison of operative management for adult acute appendicitis: open versus laparoscopic appendicectomy

Event/Total (%)*	Open (*n* = 133)	Laparoscopic (*n* = 104)	*P* value
Age (years) median, range	35 [29–51.5]	37.5 [25–53]	0.934
Female	47/133 (35)	56/104 (54)	**0.006**
No comorbidity	119/133 (90)	95/104 (91)	0.665
Time of day of operation			
8 am–6 pm	87/133 (66)	62/102 (61)	0.135
6 pm–10 pm	32/133 (24)	20/102 (20)	
10 pm–8 am	14/133 (11)	20/102 (20)	
Operative time			
< 30 min	3/133 (2)	5/102 (5)	0.612
30−60 min	53/133 (40)	44/102 (43)	
60–90 min	57/133 (43)	35/102 (34)	
90–120 min	16/133 (16)	14/102 (14)	
> 120 min	4/133 (3)	4/102 (4)	
Consultant performed procedure	49/133 (37)	33/103 (32)	0.173
Consultant performing or assisting	72/133 (54)	42/103 (41)	**0.04**
Postoperative care			
Level 2 High Dependency Unit	4/133 (3)	3/103 (3)	0.749
Level 3 Intensive Care Unit	3/133 (2)	1/103 (1)	
Converted to open from laparoscopic	–	11/104 (11)	–
Length of stay (days) median, range	3 [2–5]	2 [2–4]	**0.012**

Laparoscopic converted to open included in the laparoscopic group. 19 cases excluded due to insufficient information. Continuous data is presented as median [IQR];* p* values calculated by Mann–Whitney−*U*. Categorical data are presented as number/denominator (percentage); *p* values calculated by *χ*^2^ or Fisher’s exact test as appropriate

The bold typeset in the table highlights statistical significance

*Unless otherwise indicated

At 30-day follow-up there were no reported deaths (Table 4). The majority of patients who developed complications were in the operative management group. Intra-abdominal collections were reported in 27 (6%) cases (19 in the operative group and 5 in the antibiotics only group, *p* = 0.001). A reoperation, following initial operative intervention was required in a total of 8 patients (2%), while 6 patients (5 in the operative group), had unplanned admission to critical care. At 30 days, the overall complication rate (excluding readmissions) was 47/219 in the operative group compared to 11/242 in the conservatively managed group (*p* < 0.001).

**Table 4:** Thirty-day outcome data of initial operative versus conservative management with separate analyses for operative management versus antibiotics and interventional radiology drain placement and operative management versus antibiotics alone

Event/Total (%)	Total (*n* = 470)	Operative management (*n* = 219)	Initial conservative management (*n* = 251)			
			Antibiotics and IR drain (*n* = 251)	*P* value	Antibiotics alone (*n* = 244)	*P* value
Collection*	27/470 (6)	19/219 (8)	8/251 (4)	**0.01**	5/244 (2)	**0.001**
Collection requiring IR drain	3/470 (1)	0/219 (0)	3/251 (1)	0.252	0/244 (0)	
Collection requiring re-operation	6/470 (1)	5/219 (2)	1/251 (0)	0.102	0/244 (0)	
Ileus**	14/470 (4)	14/219 (8)	0/251 (0)	** < 0.001**	0/244 (0)	** < 0.001**
Wound infection***						
Antibiotics	18/470 (3)	16/219 (7)	2/251 (1)	** < 0.001**	2/242 (0.8)	** < 0.001**
Wound Opened	9/470 (2)	8/219 (4)	1/251 (1)	**0.014**	1/244 (0.4)	**0.02**
Hospital Acquired Pneumonia						
Oral Antibiotics	1/470 (0.2)	1/219 (0.5)	0/251 (0)	1	0/244 (0)	0.47
IV Antibiotics	6/470 (2)	6/219 (3)	0/251 (0)	**0.01**	0/244 (0)	**0.01**
Deep Vein Thrombosis/ Pulmonary Embolism	3/470 (0.2)	1/219 (0.5)	2/251 (1)	1	2/244 (1)	1
Reoperation****	8/470 (2)	7/219 (3)	1/251 (0)	**0.03**	0/244 (0)	**0.005**
Death	0/470 (0)	0/219 (0)	0/251 (0)	1	0/244 (0)	1
Unplanned level 2/3 care	6/470 (2)	5/219 (4)	1/251 (0)	0.1	1/243 (0.4)	0.11
Post presentation COVID	7/470 (1)	4/219 (2)	3/251 (1)	0.71	3/244 (1)	0.71
Failed conservative management	–	–	26/251 (10)	–	24/244 (9)	–

Categorical data are presented as number/denominator (percentage); *p* values calculated by* χ*^2^ or Fisher’s exact test as appropriate

The bold typeset in the table highlights statistical significance

*IR *Interventional radiolog placement of, *IV *Intravenous

*Collection refers to an infected fluid collection or intra−abdominal abscess requiring treatment **Ileus was defined as a partial or complete non−mechanical blockade of the small intestine ***Wound infection includes both superficial and deep incisional surgical site infection, defined according to Center for Disease Control criteria as superficial “involving only skin and subcutaneous tissues” and deep as “involving deep structures such as fascia or muscle”.**** Reoperation is defined as a return to theatre

Of those 26 (10%) managed conservatively that subsequently had an operation, 2 patients required a right hemicolectomy: one for complicated AA and the other for malignancy. Complications in those in the failed conservative management group included, 1 post-operative wound infection, 1 intra-abdominal collection, 1 unplanned and 2 planned admissions to critical care. One patient required a second operation. LOS was longer in this group than in the straight to operation group (4 days [2–5.5] vs. 3 [2–4] , *p* = 0.03).

Histology demonstrated that 214/241 (89%) of patients operated upon had acute appendicitis. Only 6 patients (3%) had a histologically normal appendix removed, with the remaining 21 (9%) having alternative pathology found at operation.

## COVID-19 and PPE

At presentation, 159 (32%) of patients were swabbed for COVID-19 with 6 (4%) positive results. Three of these patients had an operation, and 1 required reoperation. Patients managed conservatively initially were less likely to have a COVID swab (68/271 (25%), *p* < 0.001). Post-presentation, a further 4 patients that had all been swab negative on presentation, were found to have COVID. Of these, 2 had an operation. None of the 10 COVID positive patients required critical care, and there was no mortality at 30 days.

**Fig. 3:**
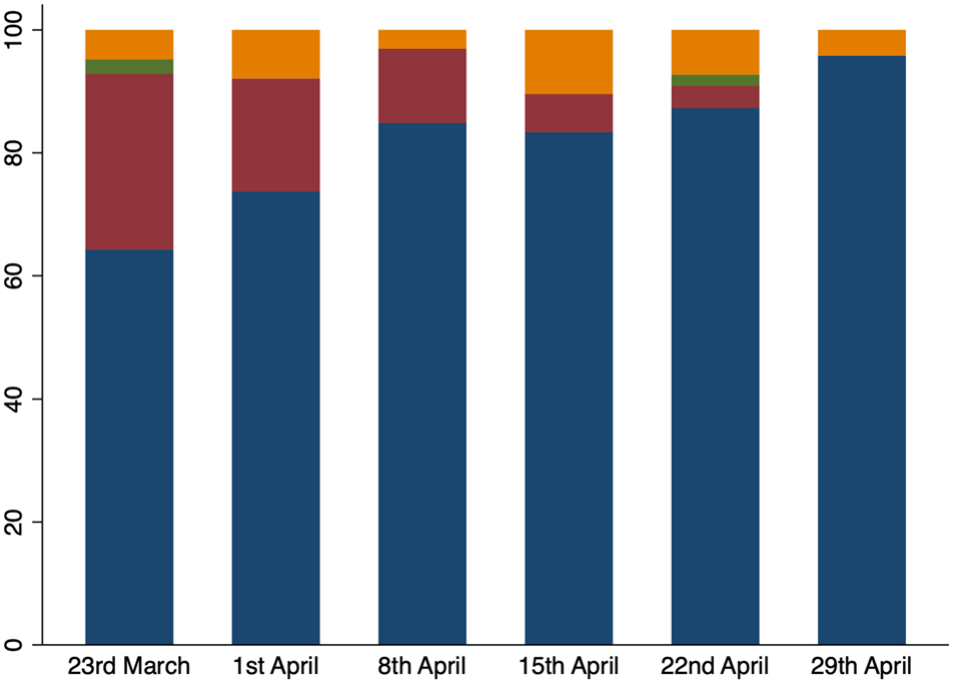
Type of Personal Protective Equipment (PPE) used when performing appendicectomy by week of the study. FFP3 mask (blue), standard surgical mask (red), none (green), and other (orange)

Through the study period use of filtering facepiece assigned protection factor 3 (FFP3) during appendicectomy increased from use in less than 60% of cases to nearly 100% (Fig. 3).

## Discussion

Disruption caused by COVID-19 has rapidly changed the management of AA in the UK, with a clear shift to conservative management. Though previously rarely used, conservative management of AA, whether with antibiotics alone or IR drain, has been effectively applied in the majority of patients in this study. With only 10% failing such management and requiring surgery, a shorter LOS and fewer complications compared to those who had initial operations, this interim analysis supports previous studies that have reported non-operative management of AA as a safe and effective option [21–26]. This study reassures surgeons about their decision making during the pandemic, supports routine CT imaging to aid such decisions and demonstrates that this option is generalisable in UK practice and perhaps, signals a reconsideration for first line treatment of AA beyond the pandemic.

### Evolution of AA management in the UK during the COVID-19 pandemic

On 20th March 2020, a leading UK surgical professional association issued COVID-19 guidance that stated “non-surgical solutions to be used to avoid surgery where possible” [17]. As of a direct result of this, normal practice in the UK of early laparoscopic appendicectomy for adult AA immediately changed to over half of patients being conservatively managed and of those having an operation, the majority having an open procedure. Strengthened by greater patient numbers having imaging, the positive short-term outcomes from conservative management support this guidance. As the pandemic evolves, professional bodies continue to provide updated guidance. This may be reflected in further management shifts that will be seen in the planned longer term follow-up of this study.

### Imaging in the diagnosis of appendicitis

Recently published work from the UK reported that almost 40% of patients had no imaging to support a clinical diagnosis of AA [8]. In contrast, our data shows 85% of patients had imaging with CT the favoured modality. Age and sex were not barriers, with CT scan very commonly used in younger patients and more frequently in women of reproductive age than before the pandemic [8]. Comparison with the recent UK observational study on right iliac fossa pain finds only 15% of their female patients had a CT scan, compared to 70% in this study [8]. In addition to aiding a decision for conservative management of AA, it is possible that clinicians wanted a definitive diagnosis of AA before embarking on an operative procedure that carried potentially higher risks for both patient and operating room staff [15]. CT is highly sensitive at detecting AA and is known to reduce the rate of negative appendicectomies significantly, making routine CT imaging perhaps with a lower radiation dose a future consideration [28]. It is not apparent why patients in this study who had an USS were significantly more likely to have conservative management. It may be that they were clinically less convincing and the USS was used to exclude other pathology (especially gynaecological) rather than confirm appendicitis. Alternatively, the sonographers may have preferred USS to make the diagnosis of AA, rather than CT scan, an approach that is recommended by the recent WSES Jerusalem Guidelines [9].

### Operative and non-operative management of AA

There were no differences between initial operative and conservative groups in the patient characteristics recorded other than age, highlighting that clinical decision-making for these patients may be multifactorial, including individual interpretation of the new guidelines, local surgical set-up for imaging and theatre access, surgical experience and patient preference. However, 9 out of 10 of those treated with antibiotics alone needed no operative intervention and stayed for a shorter time in hospital, supporting previous evidence for a non-operative strategy in AA [9, 11, 12]. For those initially conservatively managed who came to surgery, reassuringly this was a small number (10%) with the majority of decisions for surgery taken early during the admission (58% at day 2). Overall, those who failed conservative management did not have significantly poorer outcomes when compared to those having initial surgery. Although a longer LOS was reported in this group, this is to be expected as total LOS will include the conservative management days. Of the 2 right hemicolectomies performed in this group, one was for complicated appendicitis, and the other for malignancy.

The observations in this study are in keeping with previous reports of non-operative management being safe and effective for the majority of patients, with other studies reporting similar success rates, reduced social costs to the patient and less financial cost to the healthcare system if conservative management is considered as a treatment option for AA [9, 11, 12, 21–26]. However, many of these studies were conducted in a research setting, where specific inclusion criteria were applied. This contrasts sharply with our study, where all adults irrespective of their presentation, age, or CT scan findings were included.

### Laparoscopic versus open surgery

Within this study cohort, 56% (133/237) patients having an operation had an open procedure. This is at significant odds with UK practice prior to the pandemic, where only a small number of patients were having open procedures (0.4%) [8]. This is likely due to guidance issues that suggested laparoscopic surgery should be avoided due to concerns about AGPs [17, 29].

### COVID-19

The UK has been one of the hardest hit countries during the global COVID-19 pandemic, with the second highest reported mortality rate as of the beginning of June 2020 [30]. Despite this, only 32% of patients were swabbed for COVID-19 at presentation. Local policies for swabbing were not recorded and are highly likely to have varied across the UK during the early phase of the pandemic. Follow-up work may show greater rates of testing.

Only 4% tested positive for the virus with poorer outcomes not reported, irrespective of whether having surgery or conservative management. This is in contrast to recent reports of an increase in mortality after even minor to moderate surgery [16]. However, most patients with AA are young and otherwise fit, which would predispose them to better outcomes than those reported for a broad variety of operative interventions [16].

### Strengths and weaknesses

This is a large multicentre study of current AA practice in the UK with high data completion. Early analysis has allowed prompt reporting of a change in surgical practice to be demonstrated as safe in the short-term, reassuring surgeons in the current complex clinical climate and aiding decision-making in the case of a second viral wave; these findings are likely to be generalisable to other populations who normally defer to operative intervention, but whose practice has been disrupted by COVID-19, such as the United States and Europe.

The authors acknowledge that this is a pragmatic study, where bias may exist in the decision for initial management. Such bias may be a consequence of the initial conservative management group presenting with less severe symptoms compared to the initial operative group. We also appreciate that patients having laparoscopy without appendicectomy may not have been included in this data set, leading to an underestimation of the negative appendicectomy rate. One further limitation of the study is the lack of assessment of confounding variables on outcome data; this will be explored in future work with a larger dataset and maturation of follow-up to assess for longer term outcomes.

## Conclusions

The COVID-19 pandemic markedly disrupted the usual surgical management of acute appendicitis in the UK with conservative management favoured. In this setting non-operative management of AA appears to be an effective first line treatment regardless of sex, co-morbidity or frailty, with only a minority requiring surgery as a second line. Disruption allows change, and these early findings should inform the continued management of AA during the COVID-19 pandemic and perhaps beyond.

## Data Availability

Data sharing requests will be considered by the management group upon written request to the corresponding author.
